# Forthcoming Challenges in Mycotoxins Toxicology Research for Safer Food—A Need for Multi-Omics Approach

**DOI:** 10.3390/toxins9010018

**Published:** 2017-01-04

**Authors:** Luca Dellafiora, Chiara Dall’Asta

**Affiliations:** Department of Food and Drug, University of Parma, Parma 43124, Italy

**Keywords:** food toxicology, foodborne diseases, mechanisms of action, mycotoxins, risk assessment, regulations, multi-omic approach

## Abstract

The presence of mycotoxins in food represents a severe threat for public health and welfare, and poses relevant research challenges in the food toxicology field. Nowadays, food toxicologists have to provide answers to food-related toxicological issues, but at the same time they should provide the appropriate knowledge in background to effectively support the evidence-based decision-making in food safety. Therefore, keeping in mind that regulatory actions should be based on sound scientific findings, the present opinion addresses the main challenges in providing reliable data for supporting the risk assessment of foodborne mycotoxins.

## 1. Introduction: Food Toxicology Branched out from Toxicology

Toxicology has been historically defined as the “the science of poisons”. It deals with the study of the adverse health effects of chemical and physical agents on humans and animals. Actually, the term “adverse effect” indicates a wide variety of biological outcomes that may impair, at various extents, the health and well-being of the organisms undergoing exposure. Any toxic action is due to a number of modifications of homeostatic equilibria that can be observed at the different levels of complexity (e.g., whole body, organs, cellular or subcellular districts). From a molecular perspective, the primal mechanisms of toxic action commonly affect the integrity, functionality, and turnover of biological macromolecules (e.g., DNA, RNA, and proteins) or the biochemistry of the multitude of low-molecular-weight molecules (e.g., the production of reactive chemicals in cells).

Together with beneficial bioactive compounds (e.g., phytocompounds and nutraceuticals), toxicants belong to the class of xenobiotics, which groups any foreign chemical able to produce a biological effect once introduced into the body. What determines the nature of the final outcomes is not precisely defined, also on account of the fact that toxicants and healthy compounds have a number of biological targets in common. The intrinsic chemical toxicity of molecules, dose and route of exposure, and host response are among the most relevant factors for determining the final outcome, although many others can be involved (vide infra). The intrinsic toxicity mostly depends on the physicochemical properties of molecules in terms of innate chemical activity and hydrophobicity, which in turn influence a series of downstream processes, including bioavailability, bioaccessibility, clearance, and the molecular mechanisms underlying the biological action. The dose of exposure refers to how many times, how long, and to what extent the exposure to a given substance is along its lifetime. The exposure route indicates instead how a molecule may enter into the body. Finally, the host response counts all the processes along the *ADME* paradigm (i.e., the *A*bsorption, *D*istribution, *M*etabolism, and *E*xcretion of xenobiotics) that ultimately determine the amount and persistence of toxicologically active molecules at the target districts before being excreted. In particular, metabolism is responsible for a series of chemical modifications on the xenobiotics structures that, typically, cause a reduced toxicity by preventing the interaction with the biological targets and/or facilitating the excretion. Nevertheless, some metabolites may show an enhanced toxicity in respect to the parent compounds, as observed for some mycotoxins (see Section 2).

The assessment of dose–response relationship is among the most straightforward metrological parameters for the toxicological investigation. Indeed, the dose at which a xenobiotic persists in the body (intended as the amount of bioavailable toxicants) and the dose at which a given compound elicits an adverse outcome (intended as the effective concentration able to trigger the biological response) are essential factors to describe, quantify, and compare the toxic action. The toxic outcome onsets when the dose-dependent toxic damage exceeds the ability to fix the perturbation of homeostatic equilibrium. Therefore, toxicological studies address the definition of thresholds of exposure in terms of safety for humans and animals, also taking into account the combined additive, synergistic, and antagonistic effects due to the simultaneous exposure to mixtures of xenobiotics.

Food toxicology is a branch of toxicology aimed at studying the chemical substances found in food that pose a health concern upon consumption [1]. Over decades, most of the investigations in food toxicology have posed a particular emphasis on the study of toxic food constituents (i.e., toxic molecules naturally occurring within foods) and all those foreign toxicants that may enter the food chain as contaminants or additives.

Actually, foods can be counted among the most complex and rich in diversity vehicles of xenobiotics with both beneficial and adverse effects to which living organisms are exposed. Indeed, many kinds of xenobiotics may enter the food production chain at various levels. Among them, one can find man-made compounds intentionally added (e.g., food additives, ingredients, flavorings, and adulterants), or due to accidental contaminations (e.g., unwanted molecules derived from food processing or from food packaging). In addition, a huge number of xenobiotics of natural origins can occur, as well. They are grouped into all those compounds of biological origin that are actually food constituents (e.g., solanine toxin in potatoes [2] and polyphenols in some vegetables), or are accumulated in crops upon the infections by toxin-producing organisms (e.g., mycotoxins in grain-based products [3]).

The great variability, in terms of matrix complexity and food processing, affects to various extents the biological effects of foodborne compounds, thereby playing major roles also in differentiating the toxic actions. For instance, a given xenobiotic may show different bioaccessibility among diverse foods, as the changes in food composition can affect the transfer of compounds out of the food matrix during digestion (i.e., its bioaccessibility) [4]. Food processing at both industrial and domestic levels may modulate the toxicity of foodborne toxicants as well [5,6].

From a lifespan perspective, the pivotal role of secure food is beyond any doubt. Contextually, the concept of “secure food” refers to the appropriate and adequate supply of food worldwide that does not pose health issues [7]. In view of effectively tackling this forthcoming global food safety challenge, food toxicology should be framed inevitably within the context of risk assessment for food safety. In other words, investigations should try to find out answers to food-related toxicological issues, at the same time embedding the background knowledge to effectively support appropriate decision-making over the food contaminations. Indeed, it should be kept in mind that regulatory actions must be based on reliable scientific findings, although tradeoffs between public health and economic opportunities must be pursued to protect the global trade [8].

## 2. Mycotoxins and Myco-Cocktails: A Major Issue in Food Toxicology and Food Safety

Among the natural food contaminants, mycotoxins represent a major issue in food safety, and actually pose critical challenges in food toxicology. Mycotoxins are low-molecular-weight molecules produced primarily as secondary metabolites by various fungi. The most relevant fungal species involved in food contamination belong to the genera *Aspergillus, Penicillium, Fusarium, Alternaria*, and *Claviceps* [9]. Mycotoxins mainly enter the food chain worldwide as contaminants due to the pre-harvest infection of susceptible crops intended for human and animal consumption or raw materials upon noncompliant storage conditions [10,11]. Mycotoxins pose a major health concern, basically because they may elicit a number of adverse effects, and they are practically unavoidable contaminants of food and feed. Indeed, while the contaminations by man-made toxicants typically cease once the sources of contamination have been fixed, fungal infections and mycotoxins production are very hard to avoid, prevent, and control (vide infra). As a consequence, mycotoxins can be found in a huge number of food products and may represent a chronic source of contamination. Moreover, mycotoxins can be found not only in grains and grain-based foods, but also in animal-derived products as a consequence of carryover phenomena when animals are fed with contaminated feedstuffs [12]. Hence, mycotoxins may enter in several ways into the diets of many population groups, from infants to elders [13,14].

Over the centuries, mycotoxins have affected mankind in various ways and to various extents. As an example, retrospective studies have identified references in the writings of the Dead Sea Scrolls, and mycotoxins have been included among the epidemiological causes of the last of the Ten Plagues of Egypt [15]. More recently, the contamination by ergot alkaloids from *Claviceps purpurea*, which are responsible for the ergotism disease in humans [16], has been considered to be involved in the witch trials of Salem during the 17th century [17], and in the development of mystic religious movements during the Middle Ages [18]. In the modern era, mycotoxins in food have been recognized as an outstanding issue for public health in the 1960s, when almost 100,000 turkeys died due to the consumption of feed contaminated by aflatoxins from *Aspergillus flavus* [19]. Nowadays, several mycotoxins families are worldwide considered a severe threat for health and trades at a global level, and may be responsible for diseases and death, especially in the low-income countries. Among the mycotoxins, aflatoxins are of most concern due to their widespread presence and toxicity, with aflatoxin B1 being a potent carcinogen. Moreover, it has been suspected that the mycotoxins were used as bioweapon by the Soviet army in the South of Asia in the 1980s [20].

Nowadays, it is commonly accepted that the presence of mycotoxins in food raises issues for public health, as they may be involved in the onset and maintenance of several illnesses, physiological alterations, and dysfunctions [3]. Therefore, many countries have established regulations and recommendations for some mycotoxins to reduce the possible dietary exposure and protect the health and welfare of consumers. Zearalenone, deoxynivalenol, aflatoxins, fumonisins, ochratoxin A, and patulin are those regulated in the EU up to now (EC No 1881/2006, EC No 165/2010, EU No 105/2010). Actually, these represent the minority of the totality of mycotoxins potentially contaminating food. By way of example, more than 200 different molecules have been identified so far just among the trichothecenes class [21]. Moreover, the production and the accumulation in food of further unknown mycotoxins and modified forms cannot be excluded throughout.

The fungi infecting crops often produce several metabolites of a given mycotoxin, and in some cases more than one single chemical type is produced. Therefore, many parental mycotoxins and a huge number of modified forms can be found as co-contaminants in foods, merging the number of the other bioactive food constituents. The severity of crops contamination, the accumulation of mycotoxins in final products, the chemical type of mycotoxins produced, and the relative abundance of the various chemical types in the contaminated products, may vary from year to year depending on several factors, most of which are practically out of the control of human interventions [22,23].

The number and diversity of mycotoxins to which consumers are potentially exposed is even more increased by plant metabolism. Indeed, the contamination of food by mycotoxins are due to host–guest infectious processes, wherein mycotoxins may act as pathogenicity factors (by destroying a hostile environment without plan or profit) or virulence factors (thereby supporting self-defense and invasion) [24]. Plants have developed effective detoxifying systems to counteract fungal infection. They mainly reduce the amount of biologically active toxicants by activating reductive, oxidative, and conjugative transformations via phase-I and phase-II metabolic pathways. Plant metabolites can be compartmented in the edible parts of plants, thereby entering the food chain [25]. Notably, such metabolites may be the most abundant forms in the final products [26]. Nevertheless, the toxicological studies on these forms are still limited in number, and they concern mainly deoxynivalenol and zearalenone [27]. Accordingly, the toxicological relevance of plant metabolites is still very hard to assess. Such a shortage of toxicological data also impairs decision-making and inevitably causes the lack of regulation.

Also, food processing may have variable effects on both the content and chemical type of mycotoxins in the final products. Generally, it has been observed that food processing tends to reduce the content upon various degrading processes [10,28]. The different complexity of the food matrix may also affect markedly the bioaccessibility and bioavailability of xenobiotics [29]. In addition, mycotoxin byproducts with substantial chemical modifications may also arise from some food processing [30,31]. The bioactivity (toxicity) assessment of these compounds is still largely overlooked, and the hazard to health is practically unknown. Consequently, neither regulations nor recommendations are yet in force for these compounds.

Mycotoxins and modified forms (i.e., processing byproducts and metabolites of plants and fungi) are released from food matrices by the digestion process upon the consumption of contaminated food. Then, they may be transformed by the chemical conditions of digestion processes themselves or by the metabolism of the gastrointestinal microbiota [32]. After the absorption, the pool of molecules undergoes phase-I and phase-II metabolism before being excreted [27]. Metabolic modification may drastically change the toxicity of parental mycotoxins in both positive and negative directions. Typically, phase-II conjugation with sulfate groups or glucuronic acids tends to quench toxicity [25], while phase-I metabolism may produce more toxic metabolites in respect to the parent mycotoxins, as in case of alpha-zearalenol [33]. Although the chemical nature of metabolic modifications is quite conserved among the mammals, the relative abundance of the different metabolites may vary among species. Keeping in mind that some metabolites may be more toxic than the parent compounds, the predominance of more toxic metabolites over the detoxified forms plays a role in causing the species-specific susceptibility toward given mycotoxins [33].

Over the years, the scientific community involved in food safety and risk assessment have taken enormous steps forward in the identification of mycotoxins and their metabolic fate. Nevertheless, a common framework for the total and systematic identification of mycotoxin metabolites is still missing. However, the bioactivity (toxicity) assessment of metabolites is compulsory to have in-depth understanding of the mechanisms and mode of the toxic action. Indeed, the assessment of the metabolome may reveal to what extent the metabolic transformations affect the toxicodynamics of parental mycotoxins, identifying which modifications prevent the interaction with biological targets [34]. Contextually, the current shortage of toxicity data for most of mycotoxin metabolites prevents identification of which forms may mediate toxic action in vivo, and in turn, which kind of metabolic modifications are to be considered as effectively detoxifying.

Actually, the current understanding of the toxicology of foodborne mycotoxins still relies on a minority of reports, considering that only a few of the totality of mycotoxins and modified forms potentially found in food (including metabolites from mammals, plants, and fungi) have been investigated so far. Also, the toxicity of processing byproducts is still entirely unexplored. Therefore, the understanding of the mechanisms and modes of actions of foodborne mycotoxins according to the ADME paradigm is still in its infancy. On this basis, the current regulatory actions in the matter of foodborne mycotoxins, which still regulate the parental compounds only, might not comply with the real scenario in terms of safety, as regulations and recommendations might neglect some compounds of toxicological relevance that are not yet identified.

## 3. Pleiotropy of Mycotoxins Action

The adverse effects due to the dietary intake of mycotoxins can be seen at the most macroscopic level on the whole organism in both animals and humans. As an example, food contamination by zearalenone is suspected to be involved in precocious puberty in humans [35,36], and causes several sexual disorders in animals [12]. However, such macroscopic outcomes are due to an ensemble of molecular mechanisms of action that commonly involve more than one molecular target, thereby triggering a “multifactorial” toxicity (namely, pleiotropic molecular toxicity).

From a molecular perspective, any toxic event mirrors modifications at the level of integrity and functionality of DNA (genomic level), RNA (transcriptomic level), proteins (proteomic level), and/or small molecules (metabolomics level). In turn, such modifications are due to primal molecular interactions between the toxicologically active forms and the respective biological target. The precise investigation of these molecular mechanisms and how they affect cell homeostasis at the various “omic” levels is actually mandatory for the in-depth understanding of mycotoxins toxicity.

### 3.1. Multiple Mycotoxins vs Multiple Targets

The case of the *Alternaria* toxins, alternariol and congeners, provides an example of the pleiotropic action at molecular levels, wherein a single chemical type acts on multiple targets. The food contamination by *Alternaria* toxins is considered responsible for carcinogenicity at the level of the esophagus [37], and the mycotoxin alternariol is associated with a range of potential adverse health effects showing fetotoxic, teratogenic, genotoxic, mutagenic, and xenoestrogenic effects [38,39]. Genotoxic and xenoestrogenic effects arise following the interaction with at least two different molecular targets, which are topoisomerases and estrogen receptors, respectively [40,41]. In the first case, alternariol disrupts the activity of topoisomerases, which are key enzymes involved in the modulation of DNA topology [38,42], and turns them into DNA-damaging agents. In the second case, alternariol binds and activates the estrogen receptors, which are transcriptional factors under the control of estrogens, thereby triggering a xenoestrogenic stimulus in cells [39,41].

Furthermore, a given molecular target is commonly shared among several mycotoxins. For instance, the estrogen receptors can be targeted and activated also by zearalenone and some congeners [34]. Notably, the zearalenone-mediated endocrine-disrupting activity through the activation of the estrogen receptors may alter sexual behavior and impairs the functions and development of sexual apparatus, including testicular germ cell depletion, altered testis morphological parameters, reduced serum testosterone concentrations, and disturbed fertility [43]. Nevertheless, a series of other modes of action not closely linked to xenoestrogenic stimulus have been ascribed to zearalenone. Among them, one can find cytotoxicity through oxidative damage [44] and alteration of immune functionality [45], thus allowing supposition of the involvement of further molecular targets beyond the estrogen receptors.

The mechanism of action of aflatoxin represents an example of pleiotropic action, wherein the genotoxic effects are due to the direct targeting of DNA. Actually, aflatoxin B1 has been included by the International Agency for Research in Cancer (IARC) in the Group 1 carcinogens (i.e., explicit carcinogens for humans) and it is considered the most potent naturally occurring carcinogen [46]. Aflatoxins show marked hepatocarcinogenicity due to the metabolic formation of reactive epoxides that directly form DNA adducts, causing the mutations underlying carcinogenesis [47]. However, the interaction with protein targets can also be involved in the toxic activity [48,49].

### 3.2. “Omics” Toxicity of Mycotoxins

**Figure 1:**
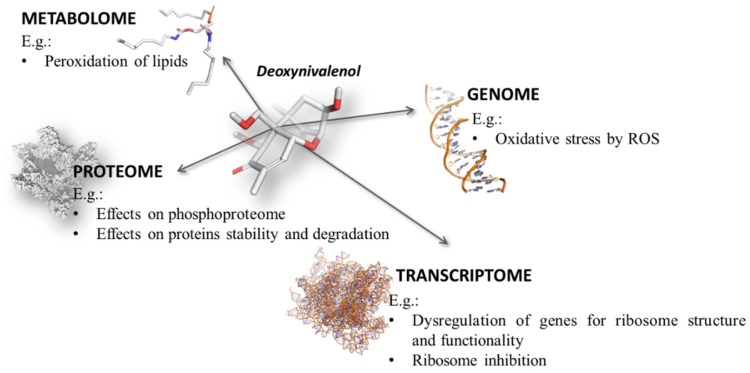
Example of the multilevel activity of deoxynivalenol.

Following the different molecular initiating events that may take place, biological effects of mycotoxins can be observed at the different “omics” levels. The family of trichothecenes, and particularly deoxynivalenol, provides an example of pleiotropy at different degrees of complexity, and over the various biological macromolecules. The crosstalk of the molecular modifications at genomic, transcriptomic, proteomic, and metabolomic levels (Figure 1) may result in a series of cellular and physiological effects. In mammals, the main toxic outcomes of deoxynivalenol on the whole organism group a huge number of dysfunctions, altering in a concentration-dependent manner the functions of gut, brain, and immune and endocrine systems [50,51] (Maresca 2013; Razafimanjato); causing modifications in the normal food and feed intake; and nutrient absorption, immunosuppression, and increased susceptibility to infection [52].

Concerning the impairment of integrity and functionality at the genomic level, deoxynivalenol has been found responsible for DNA damage and fragmentation in both in vitro and in vivo studies [53]. The genotoxic effects are mainly mediated by the production of reactive oxygen species (ROS) that may exert a nonspecific oxidative stress over all the biological macromolecules.

Deoxynivalenol has been found able to exert effects that are observable also at the transcriptomic level. For instance, it is known that the exposure to deoxynivalenol may impair immune system, and it was found to be responsible for changes in gene expression in human lymphoid cells—in particular, the upregulation of genes involved in ribosome function and structure, RNA/protein synthesis and processing, endoplasmic reticulum stress, calcium-mediated signaling, mitochondrial function, oxidative stress, T cell activation, and apoptosis [54]. 

Deoxynivalenol-dependent modifications at the proteomic level have been found as well. As an example, it was found that deoxynivalenol may act on the phosphoproteome by changing the phosphorylation pattern in terms of states and sites of many proteins of differentiated intestinal epithelial cells [55]. Effects on the glycoproteome have been observed as well, since it has been reported that deoxynivalenol, at the subtoxic doses commonly found in food and feed, may decrease the production of the mucin glycoproteins in the goblet cells by causing the reduction in the level of mRNA encoding for the intestinal membrane-associated and the secreted mucins [56]. However, it is worthy to note that the effects on proteins abundance may not be directly related to the mRNA production. As an example, it has been found that the mycotoxin dose-dependently upregulated the mRNA of the inducible NO synthase proteins in a Caco-2 model, but failed to increase production of these proteins, as protein degradation was stimulated by promoting their ubiquitinylation [57]. Conversely, generalized and nonspecific effects on protein production are mediated by the interferences with homeostasis and functionality of ribosomes. In this regard, Pan and coworkers [58] showed that deoxynivalenol induces an overall decrease in translation-related proteins that interact with the ribosome, while it increases proteins that mediate protein folding, biosynthesis, and cellular organization. The interference with ribosome activity actually represents a keystone in the deoxynivalenol toxicity. Indeed, it is known that some trichothecenes, including deoxynivalenol, targets the 60S subunit of ribosomes at the A-site and inhibits the elongation of peptide chains during transduction [52,59]. This represents the upstream molecular mechanism that may have a general effect at the proteomic levels. In addition, deoxynivalenol may also have effects on protein turnover, as low concentrations have been found responsible for changes in protein stability and degradation [52].

A direct effect of deoxynivalenol can also be observed at the level of integrity and functionality of small molecules (i.e., at the metabolomic level). For instance, the dose- and time-dependent peroxidation of lipids was observed in vitro in multiple cell lines [60,61].

### 3.3. “Omics” Methodologies for Toxicological Research

Although this review is not aimed at depicting the multiple “omics” techniques, a general overview could be useful as background. While genomic techniques—such as functional genomic, gene sequencing, and epi- or metagenomics—are often used for characterization of the fungal pathogen, and the study of the mechanisms of resistance in plants [62,63], gene expression and transcriptome analysis can provide significant insight into the mode of action of the toxic compound at the cellular level [64,65]. Among other techniques, microRNAs (miRNA) have been proven to play a crucial role in post-transcriptional regulation of genes by acting as sequence specific downregulators of already transcribed messenger RNAs (mRNAs). An increasing number of studies reported on the impact of miRNAs on the response of cells to xenobiotics, suggesting the existence of compound- and/or cell-specific miRNA response patterns. The use of tailored DNA biochips may support advances in this field [66,67]. Another challenging field of research is represented by the investigation of single nucleotide polymorphism (SNP) in humans, as a basis for the interpretation of the interindividual variability in toxicological studies, under a toxicogenomic perspective [68,69,70].

On the other hand, novel metabolite profiling techniques allowed for the identification of molecular markers’ effects in cells and/or tissues [71,72]. Finally, the implementation of metabonomics analysis of body fluids (i.e., blood, urine) may support the identification of robust biomarkers of exposure [73,74]. The integration of these techniques may therefore lead to the understanding of the effects of the human metabolism on the modulation of toxic effects [75].

In this context, high content analysis based on cell-imaging may increase the understanding of the overall changes in cellular functionality due to multiple mycotoxin exposure [76,77,78].

## 4. Combined Toxicity of Mycotoxins and Other Xenobiotics

In addition to the parental mycotoxins, several modified forms may be simultaneously found in contaminated crops and final products [79,80]. Such a wealth of compounds merges the entire spectrum of foodborne xenobiotics, which group the bioactive molecules constitutively present in foods and the huge number of contaminants. It has been shown that mycotoxin bioavailability can be affected by processes occurring upon digestion [81], or can undergo biotransformation in the human gut [32,82]. Again, they can be metabolized in the liver or in other tissues [83,84], giving rise to a wide spectrum of circulating metabolites. Therefore, the consumption of contaminated food may expose the consumers to exceptionally complex cocktails of bioactive and toxic molecules.

For many years, toxicological investigation on mycotoxins mostly carried out single-molecule studies, which allow understanding of the standalone and system-dependent potency of the various mycotoxins and modified forms. In more recent years, an increasingly growing number of in vitro studies addressed instead the combined effects of chemical mixtures. What has been observed forces a re-examination of the validity of the results obtained from single-molecule studies in terms of the health hazards within the real-world scenario. Indeed, it has been demonstrated that the biological activity of mycotoxins, in terms of dose–activity relationships, may significantly change depending on the concomitance of other bioactive compounds. Therefore, the effect of mixtures is a critical aspect to assess the potency of toxicants, and it should be taken into account to properly assess the toxicological relevance of foodborne mycotoxins. Notably, the effects of mixtures on the dose–activity relationship have been observed not only for combinations of mycotoxins, but also for mixtures of mycotoxins and other food constituents.

Concerning the first case, as example, Gao and coworkers [85] reported that the various combinations of the mycotoxins ochratoxin A, zearalenone, and alpha-zearalenol in a human intestinal cell line markedly influence the dose-dependent cytotoxicity in respect to that showed by the single toxins. In particular, the various combinations tested led to antagonistic, additive, and synergistic effects, depending on the concentrations and types of the combined mycotoxins and on the time of exposure. Among all the combinations tested, the mixture of all four mycotoxins—which can actually be found in food [86]—showed the greatest cytotoxicity. It is worthy to note that synergistic and antagonistic effects are the most commonly reported effects in the literature so far for mycotoxins mixtures [87].

Concerning the combined effects of mycotoxins and food constituents, recent findings demonstrated that the toxic activity of mycotoxins may change also when they are combined with phytocompounds [88]. Indeed, it was found that the combination of zearalenone with the phytocompound genistein—which is commonly recognized as a healthy compound [39]—showed mainly synergistic effects in triggering a xenoestrogenic response. Mixtures of alternariol and genistein showed instead both antagonistic and synergistic effects. Even if the effects were found dependent on concentrations, it has been pointed out an overall potentiating effect of mixtures, as both mycotoxins, when mixed with genistein, resulted in combinatory effects that exceeded the respective maximum activity of each single compound.

It is well known that alternariol, zearalenone, and genistein are able to trigger a xenoestrogenic response following the binding and activation of the estrogen receptors [39,89]. However, a number of foodborne polyphenols exert phytoestrogenic activity via estrogen receptor activation, and many of them may be found in food concomitantly. On this basis, it can be thought that many other polyphenols may cooperate with mycotoxins in triggering xenoestrogenic stimuli. Therefore, in the real-world conditions, the overall cooperative effects are likely much more complex than those observed in vitro for n-tuples with a limited number of elements, also considering the various transformations that may take place. Accordingly, the effective dose of a given mycotoxin that elicits toxic outcomes may change among cases, and it might strongly depend on the concomitance of other xenobiotics. Among them, the man-made food contaminants should be included as well. As an example, bisphenol A is a monomeric plasticizer used for the production of various types of plastic, including those intended for food packaging, which evokes an estrogenic response upon binding and activation of the estrogen receptors [90]. Notably, bisphenol A may migrate into foods from containers, and it can be found also in grain-based food products [91]. Hence, it might influence the overall biological effects of the eventual co-occurring mycoestrogens.

Actually, the combined effects of mixtures might influence many steps along the ADME of mycotoxins. For instance, it has been observed that dietary polyphenols may affect the absorption of mycotoxins by intestinal cells [92,93]. Also, the distribution and metabolism might be influenced as well. Indeed, the competition between mycotoxins and other foodborne xenobiotics for the plasma proteins and metabolic enzymes might have effects on several aspects, including the bioavailability in plasma, and the metabolism and clearance of the ingested mycotoxins.

On this basis, it can be thought that the same contamination level of a given mycotoxin among different foods may cause diverse effects in both qualitative and quantitative manners, depending on the totality of concomitant xenobiotics. Ultimately, this makes it hard to define tolerable levels of contamination that are truly safe for consumers. Therefore, a possible change in perspective for the definition of the thresholds of concern should be taken into consideration in the future. Indeed, it can be hypothesized that the effective concentrations of mycotoxins in eliciting adverse effects may change depending on both the food composition and overall contamination. As a consequence, a more personalized and case-specific setting of tolerable contamination levels might be necessary to ensure more secure food. For example, the tolerable limits of zearalenone might be set in a food-specific manner since the no-effect concentrations might change among diverse foods, with different contamination by other mycoestrogens and the average content of phytoestrogens.

## 5. Multi-omics Approach as the Toolbox for Unraveling Combined Toxicity

Mycotoxins in food represent a relevant threat for human health and welfare. Regulations and recommendations for some mycotoxins are enforced worldwide to set the maximum levels of contamination in food and feed, thereby reducing the dietary exposure. However, the regulatory actions among countries are still strongly influenced by the perception of the risk related to the contamination of food, instead of by objective scientific evidences only. Basically, the perception of risk depends on to what extent the countries may sustain the costs in terms of food waste and spending in the matter of public health affairs. On this basis, the entity of risk that countries are disposed to accept varies among cases, and this leads to diversified regulatory plans worldwide. The lack of status quo among countries, in terms of the tolerable levels of contamination, inevitably affects the global market and causes trade frictions, wherein developing countries are the most affected as they are hardly compliant with the contamination levels of the industrialized ones. Actually, the current state-of-the-art on mycotoxin toxicology prevents posing a solid foothold for a consensus shared globally. Therefore, it is a current duty of the Scientific Community to provide the scientific background for setting regulatory actions more precisely and effectively.

Nowadays, the toxicology of most of the mycotoxins and derived forms to which humans and animals are exposed is still unknown. In addition, the mechanisms of action underlying the known toxic actions are not still fully understood. Hence, the regulations set on the knowledge available so far might neglect some toxicologically relevant mycotoxins. This scenario cannot be sustained much longer and, therefore, in the near future, the toxicological research on foodborne mycotoxins shall provide a background of knowledge for supporting the precise setting of contamination levels that are truly safe. This means that regulatory actions shall address the mycotoxins and modified forms in food that are truly toxicologically relevant per se, or upon transformation by metabolism and processing, or upon combination with the others food components. 

**Figure 2:**
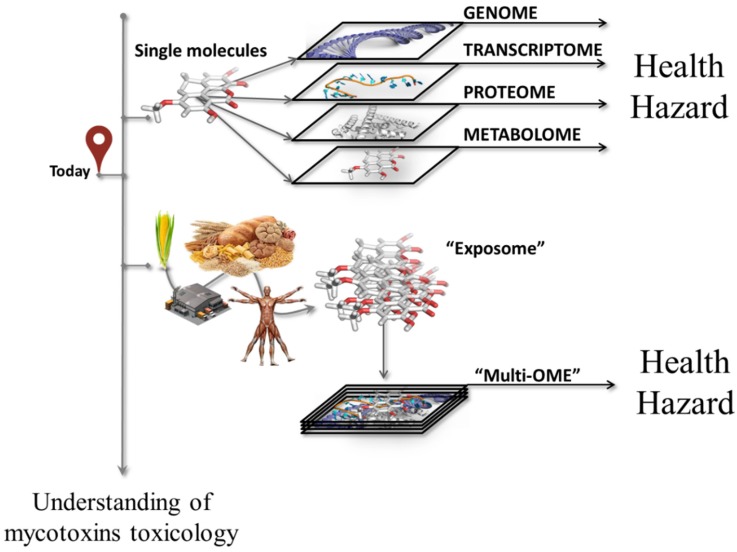
Understanding of the mechanisms of action of mycotoxins from molecular perspective.

To do this, mycotoxin toxicology should be investigated from a holistic perspective. The first step toward this direction can be the integrated use of multi-omics approach to study the molecular initiating events at a comprehensive level (Figure 2)—as, actually, the whole is more than the sum of its parts (Aristotle, 384-322 BC; attribute citations).

In other words, the multi-omics investigation should provide insights on how and to what extent the “exposome” arising from the ingestion of mycotoxins impairs functions and integrity of cells at the various omics levels. Ideally, the term “exposome” indicates the totality of the low-molecular-weight molecules that originate from the consumption of mycotoxins-contaminated foods. Such a multi-omics perspective will lead to understanding more precisely in which forms, at which dose, in which foods, and by which mechanisms mycotoxins may change molecular hemostasis. In this way, health hazards can be identified and characterized more efficiently and precisely, thereby supporting a more reliable scenario for the risk assessment of foodborne mycotoxins.

A further challenge will be to try to find out how the modifications at the multi-omics levels in the diverse cell lines correlate with the onset of injuries of tissues, organs, and apparatuses. Perhaps, this will lead to achieving, in the close future, a better understanding of mycotoxins action on the whole bodies.
